# Editorial: The role of omics characteristics in the diagnosis, treatment, and prognosis of autoimmune diseases

**DOI:** 10.3389/fimmu.2022.1069918

**Published:** 2022-11-02

**Authors:** Qinglong Wu, Kang Ning, Xiaoxiao Zhao, Feng Liao, Min Wang, Longgang Chang, Yanmin Liu, Jinmiao Chen, Ming Zhao, Zhangran Chen

**Affiliations:** ^1^ Texas Children’s Microbiome Center, Baylor College of Medicine, Houston, TX, United States; ^2^ School of Life Science and Technology, Huazhong University of Science and Technology, Wuhan, China; ^3^ Shenzhen Wedge Microbiology Research Co.Ltd, Shenzhen, China; ^4^ Inner Mongolia Shuangqi Pharmaceutical Co.Ltd, Huhhot, China; ^5^ Singapore Immunology Network (SIgN), Agency for Science, Technology and Research (ASTAR), Singapore, Singapore; ^6^ Immunology Translational Research Program, Yong Loo Lin School of Medicine, Department of Microbiology and Immunology, National University of Singapore (NUS), Singapore, Singapore; ^7^ The Second Xiangya Hospital, Central South University, Changsha, China; ^8^ Department of Gastroenterology, Zhongshan Hospital Affiliated to Xiamen University, Xiamen, China

**Keywords:** autoimmune diseases, biomarker, microRNAs, fecal microbiota, rheumatoid arthritis, omics technology

Autoimmune diseases (ADs) represent a type of disease that causes damage to the body due to the disorder of autoantigen immune tolerance and host immune responses to autoantigens. Such defects could affect any part of the body, i.e. weakening bodily function and even turning life-threatening consequences. The incidence of ADs has increased over the past decade, affecting 5% to 8% of the world population and posing great challenge to the healthcare system. Up to now, more than eighty types of autoimmune diseases have been clinically identified. In addition to protect the host against infections, cancers and other diseases, the immune system also produces elevated levels of inflammatory cytokines and auto-antibodies that could induce complex autoimmune disorders (AIDs), such as Type I diabetes (T1D), primary biliary cirrhosis (PBC), rheumatoid arthritis (RA), multiple sclerosis (MS), autoimmune liver disease (ALD), systemic lupus erythematosus (SLE), ankylosing spondylitis (AS), Sjogren syndrome (SS), and polymyositis/dermatomyositis, etc. However, the pathogenesis of ADs and many other diseases, especially certain types of cancers, still remains elusive and needs to be illuminated systematically.

This current Research Topic brings nine articles together presenting and summarizing the most recent research updates, which give us a better understanding of new potential diagnostics and therapeutics for some ADs.

With the increasing attention to the disease and the improvement of diagnostic techniques, the perception of ADs has been gradually improved. The IgG4-RD is an immune-mediated disorder with diverse autoimmune features. Wu et al. identified four major cell types and twenty-one subtypes in IgG4-RD peripheral blood monocytes (PBMCs) by using single-cell RNA-sequencing.Such findings are of great relevance for clinical characterization of IgG4-RD patients. SLE is a chronic systemic inflammatory disease that can affect many organs including kidney, lung, and skin. The CDC27 gene was uniquely found in 24 lupus lineages by using whole-exome sequencing (WES) in combination with multiple analytical methods. This work by Shang et al. revealed that CDC27 gene may serve as a biomarker for the diagnosis of SLE. Of note, WES analytics showed as a useful methodology that might screen causative genes for other diseases through small pedigrees, especially among non-close relatives. RA is an ADs characterized by chronic erosive arthritis. Using comprehensive proteomic analysis, Hu et al. discovered that Orosomucoid 1 (ORM1) in the serum is differentially expressed between healthy subjects and RA patients. ORM1 is correlated with disease activity, CD56^dim^ natural killer cell, effector memory CD8 T cell and natural killer cell in the pathological mechanism, highlighting future research on its role in RA pathogenesis. Psoriatic arthritis (PsA) is a chronic ADs characterized by both joint inflammation and skin psoriasis. Wang et al. identified differential metabolites among PsA patients, RA patients and healthy subjects by using ultra-high-performance liquid chromatography coupled with hybrid triple quadrupole time-of-flight mass spectrometry (UHPLC-Q-TOF-MS). Potential biomarkers including α/β-turmerone, glycerol 1-hexadecanoate, dihydrosphingosine, pantothenic acid and glutamine differentiate PsA from both HC and RA. Given the fact that joint inflammation occurrs in both PsA and RA patients, this study demonstrated the utility of metabolomics for early diagnosis of PsA and even for monitoring host response during treatment. Existing evidence suggests that both dysregulated innate and adaptive immune pathways contribute to the aberrant intestinal inflammatory response in patients with inflammatory bowel disease (IBD), while the exact etiology remains unclear. Using the Transcriptome and Metatranscriptome Meta-Analysis (TaMMA) framework and Cell-type Identification By Estimating Relative Subsets Of RNA Transcripts (CIBERSORT) method, Bai et al. identified and enumerated the composition of twenty-two immune cell types, demonstrating disease-specific (ulcerative colitis vs. Crohn’s disease) and lesion-specific immune cell features in IBD. These results would advance further development of precision immunotherapies for IBD.

Blood and serum circulating miRNAs have been explored as important biomarkers for early diagnosis, prognosis and prediction of drug response. Sun et al. profiled exosome miRNAs for early determination of the sensitivity of intravenous glucocorticoid therapy (ivGCs) in graves ophthalmopathy (GO) patients. Mir-885-3p was identified as a biomarker of ivGCs sensitive circulation through miRNA sequencing. This finding provides a scientific basis for the choice of treatment for GO patients and is of great clinical importance for ensuring a good prognosis in patients. Recently, accumulating studies have demonstrated that miRNAs play a key role in various cancers and ADs including RA, LSE, SS and systemic sclerosis. Chang et al. emphasized the important role of miRNA in RA susceptibility, pathogenesis and efficacy evaluation, providing a comprehensive summary to support precision medicine research in RA. Apart from conventional therapeutics, greater attention has also been focused on fecal microbiota transplantation in treating T1DM. He et al. conducted a 1-year follow-up study in two independent pediatric cohorts of T1DM disease, and found that patients in both cohorts showed better glycemic control, improved insulin resistance and no adverse effects post fecal transplantation. This study provides strong evidence for exploring fecal microbiota transplantation to treat T1DM. However, the clinical effectiveness of fecal microbiota transplantation relies on the input microbiota composition from the screened healthy donors. Interestingly, Cheng et al. found long-term travelling significantly changed the enterotype of individuals, i.e. great shifts in gut microbiota composition. Such shifts have been linked to the changes in the environment and diets that would likely account for switching their enterotypes. These results opened new avenues for probing the effects of diet and environment on human gut microbiota composition, as well as the implications of different human gut enterotypes in modelling host immunity and for treating patients with microbiota transplantation procedure.

Overall, this Research Topic provides readers with new ideas for the diagnosis and treatment of ADs. We could already understand from these works that the advancement of researches have promoted the understanding of pathogenesis of ADs. We believe the translation of these findings into clinics could largely improve the diagnosis and treatment of ADs. However, this Research Topic mainly focuses on RA, while less on other ADs. We look forward to more new studies to illustrate the disease-related mechanisms and the application of novel treatments to ADs for the health and wellness of ADs patients.

## Author contributions

QW, KN, XZ, FL, MW, LC, YL, ZC wrote and revised this article. QW, KN, JC, MZ and ZC co-edit the Research Topic. All authors made a substantial, direct, and intellectual contribution to this work and approved it for publication.

## Funding

This work is supported by grants from the National Natural Science Foundation of China (Grant No. 81900541).

## Acknowledgments

We greatly appreciate the contributions to this Research Topic by all authors and reviewers. We thank all the guest associated editors of the Research Topic, and the editorial board of the journal of Frontiers, for their support. We also appreciate Canhui Lan, from Beijing Rexinchang Biotechnology Research Institute Co. Ltd for his help in promoting this current Research Topic.

## Conflict of interest

MW, YL and ZC were employed by Inner Mongolia Shuangqi Pharmaceutical Co.Ltd. XZ, FL, LC and ZC were employed by Shenzhen Wedge Microbiology Research Co.Ltd.

The remaining authors declare that the research was conducted in the absence of any commercial or financial relationships that could be construed as a potential conflict of interest.

## Publisher’s note

All claims expressed in this article are solely those of the authors and do not necessarily represent those of their affiliated organizations, or those of the publisher, the editors and the reviewers. Any product that may be evaluated in this article, or claim that may be made by its manufacturer, is not guaranteed or endorsed by the publisher.

